# MapToGenome: A Comparative Genomic Tool that Aligns Transcript Maps to Sequenced Genomes

**Published:** 2007-02-14

**Authors:** Srikrishna Putta, Jeramiah J. Smith, Chuck Staben, S. Randal Voss

**Affiliations:** 1 Department of Biology, University of Kentucky, Lexington, KY, U.S.A; 2 Spinal Cord and Brain Injury Research Center, University of Kentucky, Lexington, KY, U.S.A

**Keywords:** comparative genomics, transcript map, linkage, gapped alignment

## Abstract

Efforts to generate whole genome assemblies and dense genetic maps have provided a wealth of gene positional information for several vertebrate species. Comparing the relative location of orthologous genes among these genomes provides perspective on genome evolution and can aid in translating genetic information between distantly related organisms. However, large-scale comparisons between genetic maps and genome assemblies can prove challenging because genetic markers are commonly derived from transcribed sequences that are incompletely and variably annotated. We developed the program MapToGenome as a tool for comparing transcript maps and genome assemblies. MapToGenome processes sequence alignments between mapped transcripts and whole genome sequence while accounting for the presence of intronic sequences, and assigns orthology based on user-defined parameters. To illustrate the utility of this program, we used MapToGenome to process alignments between vertebrate genetic maps and genome assemblies 1) self/self alignments for maps and assemblies of the rat and zebrafish genome; 2) alignments between vertebrate transcript maps (rat, salamander, zebrafish, and medaka) and the chicken genome; and 3) alignments of the medaka and zebrafish maps to the pufferfish (*Tetraodon nigroviridis*) genome. Our results show that map-genome alignments can be improved by combining alignments across presumptive intron breaks and ignoring alignments for simple sequence length polymorphism (SSLP) marker sequences. Comparisons between vertebrate maps and genomes reveal broad patterns of conservation among vertebrate genomes and the differential effects of genome rearrangement over time and across lineages.

## Introduction

Many evolutionary analyses require the identification of orthologous gene loci among the genomes of unrelated organisms. This task is not easily accomplished because genomes are rarely characterized in the same way or to the same extent. Partial and whole-genome sequences are only available for a few primary model organisms (e.g. worm, fly, pufferfish, chicken, mouse) and gene annotations vary considerably. For many more eukaryotic species, genomes are characterized by a subset of markers or transcripts that collectively constitute a physical or genetic map. The recent and continuing explosion of expressed sequence tag (EST) projects will likely increase the number of transcript maps in the next few years and these data stand to complement and enhance comparative studies. However, to exploit these resources, tools are needed to better facilitate comparisons of orthologous genes between transcript maps and whole genome sequences.

Transcript maps can be compared directly to genome assemblies by first identifying orthologous alignments between mapped transcripts and a genomic sequence, then cross-referencing the positions of orthologies. Several programs have been developed to align transcribed sequences to the genome from which they originated (e.g. Mott, 1997; Florea et al. 1998; Wheelan et al. 2001; Usuka et al. 2000; Schlueter et al. 2003; Lee et al. 2003; Ranganathan et al. 2003; Kruger et al. 2004; Wu and Watanabe, 2005; van Nimwegen et al. 2006), or to generate transcript-anchored alignments of genomic sequences among divergent taxa (e.g. Bray et al. 2003; Bray and Patcher, 2004; Yap and Patcher, 2004; Flannick and Batzoglou, 2005; Ye and Huang, 2005; Hsieh et al. 2006; Huang et al. 2006). To our knowledge no programs exist for directly aligning transcript maps to divergent genome assemblies. As in the case of within-species transcript mapping, divergent transcript-genome comparisons necessitate gapped alignments because introns often account for a significant fraction of the primary transcript, roughly 95% of the average human primary transcript (Duret et al. 1995; Venter et al. 2001). The process of aligning transcripts to divergent genomic sequence is additionally complicated by nucleotide divergence and genome-level variation (e.g. duplication and genome size evolution) that has evolved since the two species last shared a common ancestor. Precise delimitation of intron-exon boundaries is challenging for within-species mapping and nucleotide mismatches can dramatically affect alignments in the vicinity of these boundaries (Nimwegen et al. 2006). Because transcripts maps are often derived from incompletely sequenced transcripts with relatively high intrinsic error rates (e.g. ESTs), rigorous annotation of intron-exon boundaries may be impossible for many comparisons. Clearly, tools for comparing divergent transcript and genome maps must contain features that can accommodate variation in nucleotide and genome diversity parameters to optimally join gapped alignments.

A further challenge toward identifying divergent orthologs for mapped transcripts stems from the fact that they often represent only a small fraction of the transcriptome. Often, sequences are defined as orthologs if they identify each other as their highest scoring alignment, otherwise known as reciprocal best alignment orthology or monotopoorthology (reviewed by Dewey and Patcher, 2006). This strict definition of orthology is convenient for comparisons of homologous chromosome segments or complete genomes (e.g. Bray and Patcher, 2004; Borque et al. 2004; Borque et al. 2005; Woods et al. 2005; Kohn et al. 2006) because nearly all transcripts are known and comparisons can be described in terms of 1:1 relationships. However, reciprocal best orthologies are only a subset of the true orthologous relationships that are possible between two genomes. Most notably, reciprocal best orthologies may not distinguish homologs arising from duplication events, which are known to have played a major role in shaping the genomes of extant vertebrate species (e.g. Jaillon et al. 2004; Meyer and Yan de Peer, 2005; Panopoulou and Poustka, 2005; Blome et al. 2006). Because of the above factors and because substantial portions of the transcriptome are commonly missing from transcript maps, it is seemingly prudent to use a more flexible definition of orthology for aligning maps and genomes that are derived from distantly related organisms.

We developed MapToGenome as a flexible tool for aligning transcript maps and genome assemblies. MapToGenome processes sequence alignments between mapped transcripts and whole genome sequence while accounting for the presence of intronic sequences, and permits user specifiable thresholds for maximum intron length and splice site fidelity. MapToGenome defines orthology based on two user definable thresholds: minimum bitscore and the minimum ratio of an alignment’s cumulative bitscore relative to the maximum cumulative bitscore for the query genome alignment. It also cross-references mapping and genome positional information and generates oxford plots of transcript map/genome alignments. The speed and flexibility of MapToGenome permits optimization of the parameters that are used to produce gapped alignments and assign orthologies. Here we describe the operation and implementation of MapToGenome. We use datasets from several vertebrate species to illustrate the utility of MapToGenome in identifying orthologous loci among distantly related vertebrate species, including whole genome sequence from rat, chicken, zebrafish, and *Tetraodon nigroviridis,* and genetic maps for rat, *Ambystoma*, zebrafish, and medaka. We show that map-genome alignments can be improved by optimizing maximum intron length thresholds and by ignoring alignments for SSLP marker sequences. We also provide specific examples of how MapToGenome allows visualization of the correspondence of mapped genes to their presumptive genome localizations, conservation of synteny, and disruption of genomes by chromosomal rearrangements and duplications.

## Methods

### Mapping and sequence data

Sequences for whole-genome assemblies were downloaded from the University of Santa Cruz Genome Browser Gateway (http://genome.ucsc.edu/cgi-bin/hgGateway). These include genome assemblies for: rat (rn4), zebrafish (danRer4), chicken (galGal2), and *T. nigroviridis* (tetNig1). Linkage maps were obtained from the literature. The rat radiation hybrid (RH) map v3.4 (Kwitek et al. 2004) was downloaded from ftp://rgd.mcw.edu/pub/rhmap/3.4. Because many of the markers on this map correspond to only a single EST sequence, we generated a modified version for use in this study by first aligning all ESTs to the rat RefSeq database, and replacing all EST sequences with their corresponding RefSeq sequence (Supplementary Document 1). The Zebrafish RH (Geisler et al. 1999) and linkage (Gates et al. 1999; Kelly et al. 2000) maps were downloaded from the Zfin website http://zfin.org/zf_info/downloads.html#map. The salamander linkage map was from Smith et al. (2005). The medaka linkage map was generated using genotypes from Naruse et al. (2004) and the Kosambi mapping function of MapMaker QTXb20 (Supplementary Document 2).

### Sequence alignment

Similarity searches and sequence alignments were accomplished using the program BLAT (Kent, 2002). All BLAT analyses were performed using default alignment criteria and were output in NCBI blast tabular format (e.g. –out = blast8). Intraspecific comparisons of transcript and genome assembly sequence were accomplished using nucleotide/nucleotide alignments. Interspecific comparisons were accomplished using alignments between transcript sequences that were translated in three forward frames and genome sequence that was translated in six frames.

### MapToGenome

#### Algorithm

MapToGenome processes tabular alignment output from BLAT (Kent, 2002) (e.g. –out = blast8) or similarly formatted tabular alignment output, such as BLASTn (Altschul et al. 1990) (e.g. –m 8). It examines all HSPs (high-scoring segment pairs) for a given marker and generates cumulative alignments and summary statistics by summing across presumptive exons for every query-subject pair. This is achieved by consolidating otherwise continuous alignments that are interrupted by gaps (presumptive introns). By consolidation we mean: updating start and end positions for hit and query; summing identities, mismatches, gaps, and bitscores; and updating the % identity between query and hit (identities divided by the sum of identities, mismatches, and gaps). The maximum allowable length of presumptive introns (IL) and splice site fidelity (SF) are user definable. Splice site fidelity is defined as the number of base pairs that are permitted to overlap between alignments of adjacent regions of the query sequence, that have been consolidated across a presumptive intron. The algorithm works by first ordering all of the sequence alignments that have been generated for each query by their position within each chromosome of the subject genome. Next, the alignment orientation, and summary statistics are recorded for the first alignment. The orientation and alignment ends (query end and hit end for forward oriented alignments or query start and hit end for forward oriented alignments) of the first alignment are then compared to the orientation and the adjacent ends of the next alignment. If the two sequences are in the same orientation and the distance between adjacent query ends is within the SF and IL thresholds, the alignments are consolidated and compared to the next alignment. If any of these conditions is not met, consolidation is terminated and a new consolidation round is initiated.

MapToGenome also permits assignment of two user-definable thresholds for selecting the alignments that will be presented in the final alignment summary and oxford plot files. These are 1) the minimum cumulative bitscore that is necessary for an alignment to be reported, and 2) the minimum ratio of an alignment’s cumulative bitscore relative to the maximum cumulative bitscore for the query-genome alignment (i.e. proportion of best-in-genome). The program also generates an output file of all cumulative alignments prior to enforcing user-defined thresholds.

After generating a final alignment summary, MapToGenome appends information from files (provided by the user) to add the marker information and update marker/subject base positions. MapToGenome also provides a file of marker and subject positions that are concatenated across linkage groups and chromosomes, which is used for generating an oxford plot of map by genome alignments. Oxford plots are generated as postscript files.

#### Implementation

MapToGenome is written in C++ for UNIX platforms. It has been tested on Linux (2.4.x) and OS X (10.3, 10.4). It uses gnuplot (http://www.gnuplot.info) to generate oxford plots. Based on analyses of data sets ranging from several hundred to several thousand alignments, implementation of MapToGenome is limited only by memory size and processor speed. MapToGenome processed 100,438 BLAT alignments from the Rat/Chicken dataset into 4949 presumptive orthologies (at a 10 Kb intron length threshold) in <30 seconds, using a standard desktop computer [1.25 Ghz PowerPc G4 (3.2) CPU with 1GB DDR SDRAM running Mac OS X 10.3.9]. Using MapToGenome to consolidate alignments is much faster than rerunning BLAT at various gap thresholds. The initial BLAT alignment of the same Rat/Chicken dataset took 10.8 hours using a substantially more powerful computer [PowerPC G5 (3.0) CPU with 4 GB RAM running Mac OS X Server 10.3.9].

#### Availability

MapToGenome is written in C++ and is freely available to non-commercial users via web download at http://www.ambystoma.org/Tools/mapToGenome/.

#### Software Requirements

MapToGenome requires GNU make and a C++ compiler like g++ to compile the source. It uses UNIX sort command and gnuplot (http://www.gnuplot.info/) for plotting oxford grid in postscript. It also requires POSIX pipe support. This program was tested with g++ version 3.2, 3.6, gnuplot versions—3.7, 3.8, 4.0 running on Linux (2.4.x, 2.6.x), Mac OS X versions—10.2, 10.3, 10.4.

## Results and Discussion

### Within species comparisons

**Figure 1.**
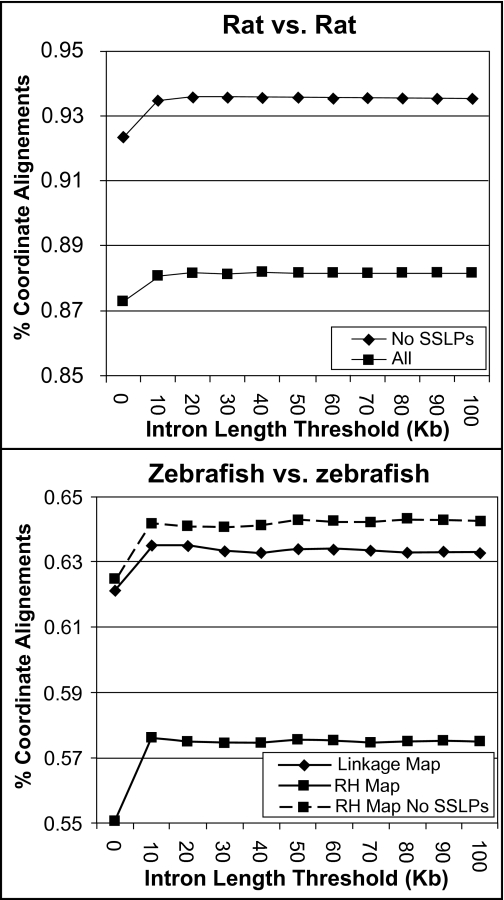
Plot of the percentage of coordinate alignments for within species comparisons of maps and whole genome assemblies. Values were calculated using several maximum intron length thresholds.

**Figure 2.**
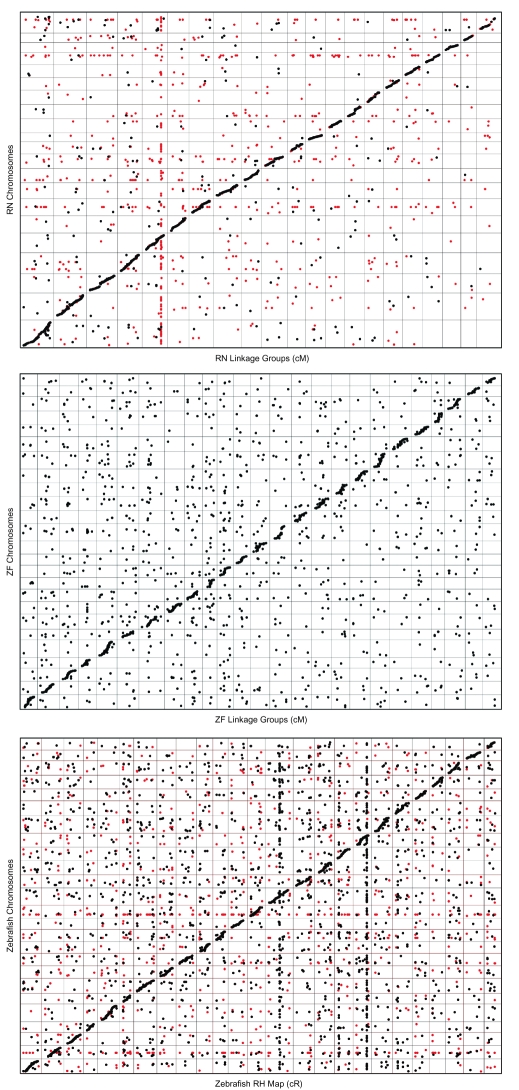
Oxford plots of alignments for within species comparisons of maps and whole genome assemblies. The rat plot was generated using a maximum intron length threshold of 20 Kb. Zebrafish plots were generated using a maximum intron length threshold of 10 Kb. Chromosomes are presented in order from left to right and bottom to top (for zebrafish: 1–25, for rat: 1–20 followed by X). Markers that are based on SSLPs are shown in red, all other markers are shown in black.

We used MapToGenome to process sequence alignments between mapped transcripts and genome assemblies of rat and zebrafish. Initially, the complete datasets were aligned using several maximum IL thresholds (ranging from 0 to 100 kb), and a SF threshold of 10 bp. In all cases, summing bitscores across presumptive introns yielded a higher proportion of coordinately aligned sequences (i.e. alignments that localize to the same chromosome between conspecific maps and genomes) relative to comparisons that do not account for intron structure (Figure 1). Furthermore, a majority of these localized to similar relative positions within chromosomes (Figure 2).

In our initial comparison of the rat RH map to the rat genome we observed a high, yet lower-than-expected proportion of coordinately aligned sequences (88.2%). In light of this, we reexamined the rat alignment data and observed that a relatively high proportion of misalignments involved markers that were developed as SSLPs. To examine the effect of these markers on the rat alignment we removed all SSLP markers from the rat map and used MapToGenome to reprocess alignments. Exclusion of SSLP markers increased the proportion of coordinately aligning sequences from 88.2% to 93.6% (Figure 1). In some cases SSLP “orthologies” formed vertical or horizontal lines in the oxford plot (Figure 2). Vertical lines represent cases where a single marker identifies a large number of orthologies that are scattered across many chromosomes, and horizontal lines represent cases where particular genomic regions tend to attract presumptively non-orthologous alignments. The patterns exhibited by SSLP markers are not particularly surprising because their genomic distribution is often strongly biased toward non-coding and repetitive regions (Arcot et al. 1995; Nadir et al. 1996; Ramsay et al. 1999; Metzgar et al. 2000; Toth et al. 2000; but see Morgante et al. 2002 for plant genomes). In general, including SSLP sequence alignments inhibited optimal alignment of the map and genome.

**Figure 3.**
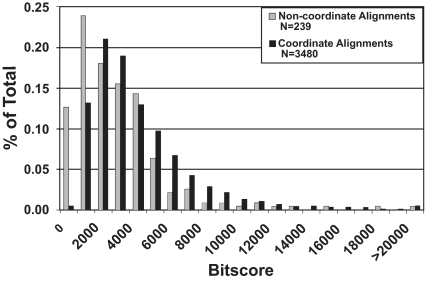
Distribution of bitscore values for coordinate and non-coordinate alignments. Values are based on alignment of the rat RH map and genome assembly, using a maximum intron length threshold of 20 Kb.

To further examine the nature of coordinately versus non-coordinately aligned transcripts, we compared the distributions of bitscore values within both classes of alignments using the 20 kb intron length threshold (Figure 3). Alignment bitscores <2,000 were overrepresented among non-coordinate alignments relative to the coordinate class, the excess corresponding to ~48 of the 239 non-coordinate alignments. Imposing an alignment bitscore cutoff of 2,000 results in a modest increase in the proportion of coordinately aligning sequences from 93.6% to 95.0%. However, this increase is achieved at the expense of 657 coordinate alignments (17.7% of the total). Thus, even at a strict bitscore threshold, ~5% of the alignments were assigned to the wrong chromosome within the rat RH map or genome assembly. Several factors likely contribute to these “errors”: 1) biological factors, such as recent gene duplicates and processed pseudogene insertions, 2) sequence and assembly errors within whole genome and transcript datasets, 3) mapping errors, and 4) failure of MapToGenome to identify “true” alignments between rat transcripts and their corresponding genome sequences. In practice, alignment “finishing” algorithms such as SPA (Nimwegen et al. 2006) could be used to improve intraspecific transcript/genome alignments that are generated by BLAT. However, SPA does not currently permit integration of linkage or RH mapping data, or tabular output formatting.

By comparison to the rat dataset, genetic maps for zebrafish show much less correspondence to the zebrafish genome assembly. At intron length thresholds of 10 and 20 kb, only 63.5% of sequence alignments mapped to coordinate chromosomes within the zebrafish linkage map and genome assembly. The proportion of coordinately aligned sequences was 57.6% when SSLP markers were included in the dataset and 64.3% when SSLP markers were excluded (Figure 1). As was observed for the rat dataset, the lower proportion of coordinately aligning sequences within the complete RH dataset suggests that inclusion of SSLP mapping data into genome alignments may inhibit optimal alignment of maps and assemblies. Notably, low proportions of coordinately aligning transcripts (~64%) were observed for comparisons between the zebrafish genome assembly and both zebrafish genetic maps. This replicated observation suggests that a substantial proportion of zebrafish genes are not currently localized to the correct chromosome within the zebrafish (Zv6) genome assembly.

Inspection of the zebrafish Oxford plots reveals another conspicuous difference between the RH map and linkage group based comparisons. Specifically, the slopes of the lines that are created by coordinate alignments are less linear within the zebrafish linkage map/genome plot. This nonlinear relationship between linkage distance and physical distance is expected because linkage distances are based on recombinational frequencies, rather than physical distance. Regions of the oxford plot with steeper slopes presumably represent regions of the genome wherein recombinational distance changes at a relatively slower rate (e.g. suppression of recombination near the centromeres) and regions of the oxford plot with gentler slopes presumably represent regions of the genome wherein recombinational distance changes at a relatively faster rate (e.g. near the telomeres) (Jensen-Seaman et al. 2004). It is therefore important to consider not only the relative position of orthologies within chromosomes, but also the relationship between map units and physical distance, when interpreting the Oxford plots that are output by MapToGenome.

### Comparing divergent genomes

Although self/self comparisons are useful for evaluating genome maps and assemblies, comparisons between divergent genomes are more interesting in an evolutionary context. Furthermore, these comparisons provide a measure of the cross predictability of gene location information between species and a means of cross-referencing gene location information between highly developed whole genome assemblies and less developed genetic maps.

In order to evaluate between-species alignments, we used the association index λ as a measure of the conservation of broad sense synteny (i.e. the distribution of genes among chromosomes) between species. The λ index measures the extent to which knowing the location of genes in either of two species is predictive of their location in the other (Goodman and Kruskal, 1954; Housworth and Postlethwait, 2002; Smith and Voss, 2006). In probabilistic terms, the λ index measures “the relative decrease in probability of erroneous guessing” (Goodman and Kruskal, 1954) that is gained by knowing the position of an ortholog in either of two species. For example, a λ value of 0.5 means that knowing the location of an ortholog in either of two species results in a 50% decrease in the probability of incorrectly guessing its location in the other species, or doubles the probability of correctly guessing its location. For this study, we primarily used the λ index to evaluate orthology calls based on different IL thresholds. Errors in assigning orthology should tend to decrease λ because they will cause an ortholog to be assigned to the wrong chromosome, and hence obscure associations that have been conserved through time. When the λ index is estimated using accurate orthology assignments it provides a measure of the extent to which the inter-chromosomal distribution of genes has been conserved between two species, or the conservation of broad-sense synteny. Because this measurement is interpretable in a probabilistic sense, λ values can be informatively compared between independent studies. The same is not true for similar chi-square based statistics (Fisher, 1938; Blalock, 1958; Goodman and Kruskal, 1954; Kendall and Stuart, 1967).

#### Vertebrates vs. chicken

**Figure 4.**
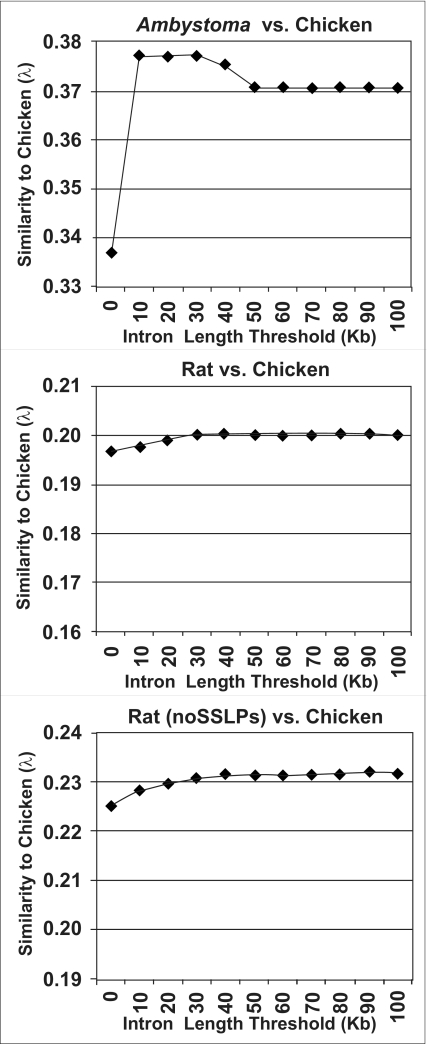
Plot of the λ values for comparisons between tetrapod (*Ambystoma*, and rat) genetic maps and the chicken genome assembly. Values were calculated using several maximum intron length thresholds.

**Figure 5.**
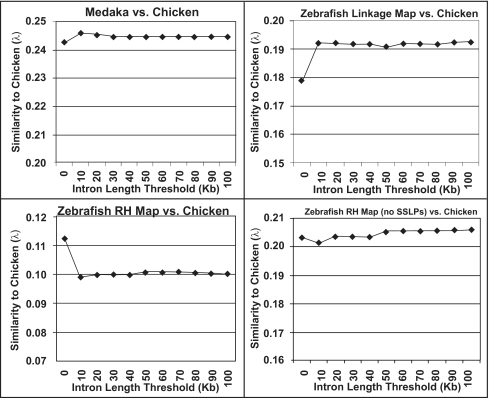
Plot of the λ values for comparisons between fish (medaka, and zebrafish) genetic maps and the chicken genome assembly. Values were calculated using several maximum intron length thresholds.

We aligned genetic maps for one representative mammal (rat), one representative amphibian (*Ambystoma*), and two fish (zebrafish and medaka) to a representative reptile (chicken). These comparisons span divergence times of ~310 million years (MY) (rat/chicken), ~370 MY (*Ambystoma*/chicken), and ~450 MY (fish/chicken) (Kumar and Hedges, 1998). Maps were aligned to the chicken genome using the same initial IL and SF thresholds that were used for self/self alignments. In general, gapped alignments yielded higher values of λ, although the maximum intron length threshold that maximized λ varied among alignments (Figures 4 and 5).

**Figure 6.**
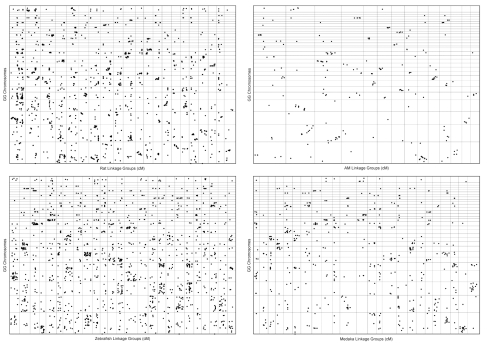
Oxford plots of vertebrate/chicken alignments. Maximum intron length thresholds of 10, 30, 90, and 100 Kb were used for the medaka, *Ambystoma* (AM), rat, and zebrafish alignments, respectively. Chromosomes and linkage groups are presented in order from left to right (for zebrafish: 1–25; for rat: 1–20 followed by X; for *Ambystoma*: 1–14; for medaka: 1–24). Chicken chromosomes are presented in order from bottom to top, 1–32, followed by W and Z. Chicken chromosomes 25, and 29–31 are omitted, these chromosomes are not represented in the current chicken genome assembly (v2.1).

Estimation of λ can provide a measure of the cross-predictability of chromosomal assignments. However, smaller chromosomal segments, rather than entire chromosomes, are often the functional units of conservation. Conserved chromosomal segments can be identified as discreet clusters of points within oxford plots. Statistical methods exist for identifying chromosomal segments with highly conserved gene orders (Calabrese et al. 2003), although these methods have not been fully adapted for use in multi-chromosomal genomes (Smith and Voss, 2006). The degree to which the linear order of segments has been conserved can also be assessed by directly inspecting oxford plots (Figure 6). Highly conserved segments can be visualized as semi-linear clusters of points, whereas segments within which linear order has been less conserved appear as unordered clusters. In comparison to the fish/chicken plots, orthologies appear to be more tightly clustered and somewhat more linear in rat/chicken and *Ambystoma*/chicken plots. This presumably reflects the greater divergence time spanned by the fish/chicken comparison, during which intrachromosomal rearrangements have accumulated.

#### Fish vs. fish

We also compared the whole genome sequence of *Tetraodon* to three fish maps: the medaka linkage map, the zebrafish linkage map, and the zebrafish RH map. Comparisons between fish linkage maps and the *Tetraodon* genome can permit better prediction of the chromosomal location of large numbers of *Tetraodon* gene orthologs within the genomes of less developed fish species. These comparisons can also provide insight into the structure of the ancestral teleost genome, which will lend a critical outgroup perspective for interpreting patterns of conservation and changes that are observed among amphibian and amniote vertebrates (e.g. Smith and Voss, 2006).

**Figure 7.**
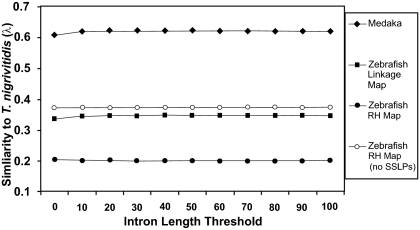
Plot of the λ values for comparisons between fish (medaka, and zebrafish) genetic maps and the *T. nigroviridis* genome assembly. Values were calculated using several maximum intron length thresholds.

The zebrafish/*T. nigroviridis* comparisons span a large fraction of the history of the euteleostei (~110–160 MY) whereas the medaka/*T. nigroviridis* comparison spans ~60–80 MY (Nelson, 1994; Wittbrodt et al. 2002). As was observed for the above comparisons, gapped alignments yielded larger λ values for comparisons between linkage maps and the *T. nigroviridis* genome (Figure 7). Values of λ that were estimated for the medaka/*T. nigroviridis* were substantially higher than those estimated for the zebrafish/*T. nigroviridis* comparison. Presumably this difference is due in part to the additional ~60 MY of shared ancestry between medaka and *T. nigroviridis*.

In a pattern similar to comparisons between the zebrafish RH map and genome, comparisons between the zebrafish RH map and the *T. nigroviridis* genome yielded substantially lower values of λ relative to comparisons that used the zebrafish linkage map (Figure 7). Removing SSLP markers from the RH dataset resulted in an overall increase in estimated λ values, yielding values that were similar to, though slightly higher than, those based on the zebrafish linkage map. Notably, several of the existing fish genetic maps consist largely of SSLP based markers, and these SSLP markers have served as the basis for numerous comparative genetic studies (e.g. Woram et al. 2004; Danzmann et al. 2005; Lee et al. 2005; Gharbi et al. 2006; Stemshorn et al. 2006). Our general observation that excluding SSLP markers results in higher cross-predictability (% coordinate alignments and λ) indicates that caution should be exercised when interpreting comparisons that are based on SSLP marker data.

**Figure 8.**
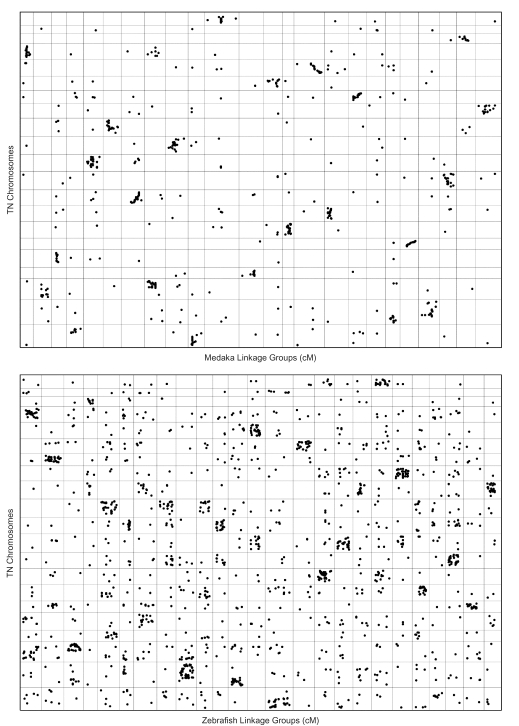
Oxford plots of Fish/*T. nigroviridis* alignments. A maximum intron length thresholds of 60 Kb was used for both alignments. Chromosomes and linkage groups are presented in order from left to right (for zebrafish: 1–25; for medaka: 1–24). *T. nigroviridis* chromosomes are presented in order from bottom to top, 1–21.

Oxford plots of fish/fish comparisons provide further insight into the evolution of fish genomes. The oxford plot for medaka/*T. nigroviridis* reveals that the structure of these genomes has been highly conserved since these two species last shared a common ancestor (Figure 8). Many medaka/*T. nigroviridis* chromosomes show strong evidence of conserved synteny. Furthermore, many of these orthologs lie in nearly diagonal clusters, indicating higher order conservation of chromosome segments. Oxford plots for zebrafish/*T. nigroviridis* reveal several dense clusters of syntenies that are consistent with widespread conservation of synteny, although there appears to be very little conservation of the large-scale linear order of orthologs. Thus, the zebrafish/*T. nigroviridis* comparison reveals a greater extent of interchromosomal and intrachromosomal rearrangement relative to the medaka/*T. nigroviridis* comparison, although the general chromosomal location of orthologs is apparently highly predictable on both species when compared to the *T. nigroviridis* assembly.

## Summary

We developed MapToGenome as a flexible tool for aligning transcript maps and genome assemblies. The speed and flexibility of MapToGenome permits optimization of the parameters that are used to produce gapped alignments and assign orthologies. We show that, in general, map/genome alignments can be improved by optimizing maximum intron length thresholds, and by ignoring alignments for SSLP marker sequences. We also showed that MapToGenome is a useful tool because it allows visualization of: 1) the correspondence of mapped genes to their presumptive genomic localizations, 2) differential scaling of recombinational and physical map distances, 3) conservation of synteny, and 4) disruption of genomes by chromosomal rearrangements and duplications.

## References

[b1-ebo-03-15] Altschul SF, Gish W, Miller W (1990). Basic local alignment search tool. J. Mol. Biol.

[b2-ebo-03-15] Arcot SS, Wang Z, Weber JL (1995). *Alu* repeats: a source for the genesis of primate microsatellites. Genomics.

[b3-ebo-03-15] Blomme T, Vandepoele K, De Bodt S (2006). The gain and loss of genes during 600 million years of vertebrate evolution. Genome Biol.

[b4-ebo-03-15] Blalock HM (1958). Probabilistic interpretations for the mean square contingency. J. Am. Stat. Assoc.

[b5-ebo-03-15] Bourque G, Pevzner PA, Tesler G (2004). Reconstructing the genomic architecture of ancestral mammals: lessons from human, mouse, and rat genomes. Genome Res.

[b6-ebo-03-15] Bourque G, Zdobnov EM, Bork P (2005). Comparative architectures of mammalian and chicken genomes reveal highly variable rates of genomic rearrangements across different lineages. Genome Res.

[b7-ebo-03-15] Bray N, Pachter L (2004). MAVID: constrained ancestral alignment of multiple sequences. Genome Res.

[b8-ebo-03-15] Bray N, Dubchak I, Pachter L (2003). AVID: A global alignment program. Genome Res.

[b9-ebo-03-15] Calabrese PP, Chakravarty S, Vision TJ (2003). Fast identification and statistical evaluation of segmental homologies in comparative maps. Bioinformatics.

[b10-ebo-03-15] Danzmann RG, Cairney M, Ferguson MM (2005). A comparative analysis of the rainbow trout genome with two other species of fish (Arctic char and Atlantic salmon) within the tetraploid derivative Salmonidae family (subfamily: Salmoninae). Genome.

[b11-ebo-03-15] Dewey CN, Pachter L (2006). Evolution at the nucleotide level: the problem of multiple whole-genome alignment. Hum. Mol .Genet.

[b12-ebo-03-15] Duret L, Mouchiroud D, Gautier C (1995). Statistical analysis of vertebrate sequences reveals that long genes are scarce in GC-rich isochors. J. Mol. Evol.

[b13-ebo-03-15] Fisher RA (1938). Statistical Methods for Research Workers.

[b14-ebo-03-15] Flannick J, Batzoglou S (2005). Using multiple alignments to improve seeded local alignment algorithms. Nucleic Acids Res.

[b15-ebo-03-15] Florea L, Hartzell G, Zhang Z (1998). A computer program for aligning a cDNA sequence with a genomic DNA sequence. Genome Res.

[b16-ebo-03-15] Gates MA, Kim L, Egan ES (1999). A genetic linkage map for zebrafish: comparative analysis and localization of genes and expressed sequences. Genome Res.

[b17-ebo-03-15] Geisler R, Rauch GJ, Baier H (1999). A radiation hybrid map of the zebrafish genome. Nat. Genet.

[b18-ebo-03-15] Gharbi K, Gautier A, Danzmann RG (2006). A linkage map for brown trout (*Salmo trutta*): chromosome homeologies and comparative genome organization with other Salmonid fish. Genetics.

[b19-ebo-03-15] Goodman LA, Kruskal WH (1954). Measures of association for cross classifications. J. Am. Stat. Assoc.

[b20-ebo-03-15] Housworth EA, Postlethwait J (2002). Measures of synteny conservation between species pairs. Genetics.

[b21-ebo-03-15] Hsieh SJ, Lin CY, Liu NH (2006). GeneAlign: a coding exon prediction tool based on phylogenetical comparisons. Nucleic Acids Res.

[b22-ebo-03-15] Huang W, Umbach DM, Li L (2006). Accurate anchoring alignment of divergent sequences. Bioinformatics.

[b23-ebo-03-15] Jaillon O, Aury JM, Brunet F (2004). Genome duplication in the teleost fish *Tetraodon nigroviridis* reveals the early vertebrate proto-karyotype. Nature.

[b24-ebo-03-15] Jensen-Seaman MI, Furey TS, Payseur BA (2004). Comparative recombination rates in the rat, mouse, and human genomes. Genome Res.

[b25-ebo-03-15] Kelly PD, Chu F, Woods IG (2000). Genetic linkage mapping of zebrafish genes and ESTs. Genome Res.

[b26-ebo-03-15] Kendall MG, Stuart A (1967). The Advanced Theory of Statistics: Volume 2 inference and relationship.

[b27-ebo-03-15] Kent WJ (2002). BLAT—The BLAST-like alignment tool. Genome Res.

[b28-ebo-03-15] Kohn M, Hogel J, Vogel W (2006). Reconstruction of a 450-MY-old ancestral vertebrate protokaryotype. Trends Genet.

[b29-ebo-03-15] Kruger J, Sczyrba A, Kurtz S (2004). e2g: an interactive web-based server for efficiently mapping large EST and cDNA sets to genomic sequences. Nucleic Acids Res.

[b30-ebo-03-15] Kwitek AE, Gullings-Handley J, Yu J (2004). High-density rat radiation hybrid maps containing over 24,000 SSLPs, genes, and ESTs provide a direct link to the rat genome sequence. Genome Res.

[b31-ebo-03-15] Lee BY, Lee WJ, Streelman JT (2005). A second-generation genetic linkage map of tilapia (Oreochromis spp.). Genetics.

[b32-ebo-03-15] Metzgar M, Bytof J, Willis C (2000). Selection against frameshift mutations limits microsatellite expansion in coding DNA. Genome Res.

[b33-ebo-03-15] Meyer A, Van de Peer Y (2005). From 2R to 3R: evidence for a fish-specific whole genome duplication (FSGD). BioEssays.

[b34-ebo-03-15] Morgante M, Hanafey M, Powell W (2002). Microsatellites are preferentially associated with nonrepetitive DNA in plant genomes. Nature Genet.

[b35-ebo-03-15] Mott R (1997). EST_GENOME: a program to align spliced DNA sequences to unspliced genomic DNA. Comput. Appl. Biosci.

[b36-ebo-03-15] Nadir E, Margalit H, Gallily T (1996). Microsatellite spreading in the human genome: evolutionary mechanisms and structural implications. Proc. Natl. Acad. Sci., U.S.A..

[b37-ebo-03-15] Naruse K, Tanaka M, Mita K (2004). A medaka gene map: the trace of ancestral vertebrate proto-chromosomes revealed by comparative gene mapping. Genome Res.

[b38-ebo-03-15] Nelson JS (1994). Fishes of the world.

[b39-ebo-03-15] Panopoulou G, Poustka AJ (2005). Timing and mechanism of ancient vertebrate genome duplications—the adventure of a hypothesis. Trends Genet.

[b40-ebo-03-15] Ramsay L, Macaulay M, Cardle L (1999). Intimate association of microsatellite repeats with retrotransposons and other dispersed repetitive elements in barley. Plant J.

[b41-ebo-03-15] Smith JJ, Putta S, Walker JA (2005). Sal-Site: integrating new and existing ambystomatid salamander research and informational resources. BMC Genomics.

[b42-ebo-03-15] Smith JJ, Voss SR (2006). Gene order data from a model amphibian (*Ambystoma*): new perspectives on vertebrate genome structure and evolution. BMC Genomics.

[b43-ebo-03-15] Stemshorn KC, Nolte AW, Tautz DA (2005). Genetic Map of *Cottus gobio* (Pisces, Teleostei) based on microsatellites can be linked to the Physical Map of *Tetraodon nigroviridis*. J. Evol. Biol.

[b44-ebo-03-15] Toth G, Gaspari Z, Jurka J (2000). Microsatellites in different eukaryotic genomes: survey and analysis. Genome Res.

[b45-ebo-03-15] van Nimwegen E, Paul N, Sheridan R (2006). SPA: a probabilistic algorithm for spliced alignment. PLoS Genet.

[b46-ebo-03-15] Venter JC, Adams MD, Myers EW (2001). The sequence of the human genome. Science.

[b47-ebo-03-15] Wittbrodt J, Shima A, Schartl M (2002). Medaka—a model organism from the far East. Nat. Rev. Genet.

[b48-ebo-03-15] Woods IG, Wilson C, Friedlander B (2005). The zebrafish gene map defines ancestral vertebrate chromosomes. Genome Res.

[b49-ebo-03-15] Woram RA, McGowan C, Stout JA (2004). A genetic linkage map for Arctic char (*Salvelinus alpinus*): evidence for higher recombination rates and segregation distortion in hybrid versus pure strain mapping parents. Genome.

[b50-ebo-03-15] Wu TD, Watanabe CK (2005). GMAP: a genomic mapping and alignment program for mRNA and EST sequences. Bioinformatics.

[b51-ebo-03-15] Yap VB, Pachter L (2004). Identification of evolutionary hotspots in the rodent genomes. Genome Res.

[b52-ebo-03-15] Ye L, Huang X (2005). MAP2: multiple alignment of syntenic genomic sequences. Nucleic Acids Res.

